# Impact of an Omega-3-Enriched Sheep Diet on the Microbiota and Chemical Composition of Kefalograviera Cheese

**DOI:** 10.3390/foods11060843

**Published:** 2022-03-15

**Authors:** Athina Tzora, Aikaterini Nelli, Chrysoula (Chrysa) Voidarou, Konstantina Fotou, Eleftherios Bonos, Georgios Rozos, Katerina Grigoriadou, Panagiotis Papadopoulos, Zoitsa Basdagianni, Ilias Giannenas, Ioannis Skoufos

**Affiliations:** 1 Laboratory of Animal Health, Food Hygiene and Quality, Department of Agriculture, University of Ioannina, 47132 Arta, Greece; knelli@uoi.gr (A.N.); xvoidarou@uoi.gr (C.V.); kfotou@uoi.gr (K.F.); ebonos@uoi.gr (E.B.); clevervet@hotmail.com (G.R.); ppapadopoulos@uoi.gr (P.P.); jskoufos@uoi.gr (I.S.); 2 Institute of Plant Breeding and Genetic Resources, Hellenic Agricultural Organization—DEMETER, Thermi, 57001 Thessaloniki, Greece; kgrigoriadou@ipgrb.gr; 3 Laboratory of Animal Husbandry, Department of Animal Production, School of Agriculture, Aristotle University of Thessaloniki, 54124 Thessaloniki, Greece; basdagianni@agro.auth.gr; 4 Laboratory of Nutrition, School of Veterinary Medicine, Faculty of Health Sciences, Aristotle University of Thessaloniki, 54124 Thessaloniki, Greece; igiannenas@vet.auth.gr

**Keywords:** Kefalograviera cheese, sheep, milk, ω-3 enriched diets, MALDI-TOF MS, microbiota

## Abstract

Kefalograviera is a well-known hard Greek cheese. The aim of this study was to determine how milk produced from ewes fed omega-3-enriched diets could influence the microbiota as well as the chemical composition of Kefalograviera cheese. At the start of the trial, 30 dairy ewes (Lesvos and Chios crossbreed) were selected and fed a conventional diet, based on alfalfa hay, straw and concentrate feed that contained soybean meal for a period of thirty days. Then, for a period of sixty days the same ewes were fed an omega-3-enriched concentrate feed with a lower level of soybean meal that contained 10% flaxseed and 10% lupins. Milk yield was collected individually on Days 30, 60 and 90 and used to produce three different batches of Kefalograviera cheeses, at the same cheese factory, by using a traditional recipe and identical preparation conditions (pasteurization of milk, salt, rennet and culture). Sample analysis was done after six months of Kefalograviera cheese ripening. MALDI-TOF-MS (matrix-assisted laser desorption/ionization time of flight mass spectrometry) identification was performed by contrasting the samples’ mass spectra with the corresponding reference database. The correlation between the different Kefalograviera cheeses revealed the predominant species being *Lactococcus lactis, Lactobacillus rhamnosus, Lactobacillus plantarum, Lactobacillus brevis*, *Lactobacillus paracasei*, *Enterococcus faecium* and *Enterococcus faecalis*, with significant quantitative differences between the experimental groups and the controls. *Pediococcus* spp. was isolated only from the experimental groups’ cheeses and *Staphylococcus* spp. only from the controls’ cheese, suggesting—among other differences—a bacterial microbiota distinction between the groups. Moreover, increased levels of alpha-linolenic acid and total polyunsaturated omega-3 fatty acids were noted in the enriched Kefalograviera cheeses. These promising findings suggest that enriched Kefalograviera cheese could be manufactured via enriching the ewes’ diets, with potential benefits for the consumers’ health.

## 1. Introduction

Kefalograviera is a traditional Greek cheese classified as Protected Destination of Origin (PDO). It is produced in the areas of Western Macedonia, Epirus, Aitoloakarnania and Evritania, mainly from ewes’ milk and smaller amounts of goats’ milk (up to 10% *v*/*v*) [1]. Recent papers show that it is becoming one of the most widespread cheeses in Greece, with annual production of around 3000 tons [2]. The ripening period commercially is at least three months, but Kefalograviera cheese can be kept for a year to achieve better quality and organoleptic characteristics and the final product is a hard, low-moisture cheese (less than 40%), with a minimum dry fat content of 40%. It is sold in wheels or wedges and is easily identified by its firm texture and light brown rind. It is considered a white to yellowish full fat cheese with a salty and piquant flavour and rich aroma. These organoleptic characteristics are very attractive; thus, Kefalograviera has become a very popular cheese to consumers [3]. The flavour development in hard cheeses such as Kefalograviera is affected by volatile compounds derived from biochemical procedures such as lipolysis, proteolysis and glycolysis [4]. Kefalograviera cheese can be a profitable dairy product because of its authenticity as a PDO product and its “full of nutrients” composition.

Feeding management of the sheep whose milk is fermented to Kefalograviera cheese can affect this milk’s composition and therefore the quality and quantity of cheese. Thus, it can be advantageous in the cheese manufacturing process [5,6,7]. Additionally, the nutritional value factor of the cheese is related to a higher level of fatty acids, especially of omega-3 fatty acids, which are considered beneficial for consumers’ health [8]. It has been noted that, the higher concentration of omega-3 fatty acids in ruminant feed, the more the dairy products’ composition is affected [9]. It is a fact that the increasing attention of consumers to the nutritional and health aspects of food has created a tendency among sheep farmers and dairies to produce milk and dairy products, respectively, with a modified lipid composition. The fat concentration in milk can be altered by diet, especially by factors affecting rumen fermentation [10,11].

On the other hand, the high concentration of Saturated Fatty Acids (SFA) in milk’s fat is considered as a health hazard because the increased daily intake of such fatty acids could increase the blood cholesterol level, which, in turn, is associated with an increased risk of cardiovascular disease. There is a consensus in the scientific literature that cheese contains lipid compounds that can improve consumers’ health, such as conjugated linolenic acid (CLA), oleic acid (OA), and vaccenic acid (VA) [12,13,14]. Handling of the fatty acid profile of milk, through nutritional interventions to the sheep, aiming to improve the fat content of dairy products, is a significant priority for the dairy industry [10]. The reason for this strategy is that certain fatty acids of the milk fat, such as butyric acid (C4:0), oleic acid, *n*-3 and *n*-6 polyunsaturated fatty acids, *trans*-vaccenic acid (*trans*11 C18:1), and conjugated linoleic acid *cis*9-*trans*11 C18:2 (CLA), are considered as beneficial to human health [15].

Some bacterial species are capable of producing omega-3 fatty acids [16]. Bianchi et al. (2014) isolated 40 Antarctic bacterial species producing either docosahexaenoic acid (DHA) or eicosapentaenoic acid (EPA) [17]. *Galactomyces geotrichum* moulds showed the ability for production of n-6 and n-3 PUFA and when added to prepared cheese was rich in omega-3 fatty acids [18].

Going one step further, a pivotal role in cheese qualitative characteristics is also played by the cheese-associated microbiota by metabolizing carbohydrates and proteins during the ripening procedures [19]. Cheese microbiota can be influenced by the physicochemical milk’s compounds and linked to several sheep feeding-related and geographical location factors [20,21]. Lactic acid bacteria in non-starter cultures have the ability to increase the bioactive fatty acids content in sheep cheeses. For this purpose, CLA-producing adjunct cultures are a practical solution to improve the nutritional value of cheese fat towards a healthier direction. CLA-producing *Lactobacilli* do not alter the sensory characteristics of cheese. Moreover, ripening time can potentially affect the fatty acid content [22].

Despite these advances, most research has been focussing on the effect of omega-3 in the feeds to the content of omega-3 in the cheese and few papers have discussed the effect of omega-3 in the modification of the microflora of the cheese. Most researchers agree that grazing pastures is an excellent source of dietary omega-3, but in the Mediterranean environment, green pastures last for a few months, usually from November till May; it is, therefore, necessary to feed concentrated diets rich in these substances. The aim of this study is not only to investigate the effect of enriching ewes’ diets with omega-3 fatty acids originating from a combination of concentrated foods to the content of omega-3 fatty acids in produced Kefalograviera, but also the effect of this enrichment in omega-3 fatty acids to the core microbiota of the cheese, analysed by high-throughput technology of Matrix-Assisted Laser Desorption/Ionization Time-of-Flight Mass Spectrometry (MALDI-TOF MS). The present study is the first—to our knowledge—investigating these effects.

## 2. Materials and Methods

### 2.1. Study Design

#### 2.1.1. Animals and Dietary Treatments

The experiment was performed in a sheep rearing farm (Vonitsa, Aitoloakarnania, Greece). At the start of the trial, 30 dairy ewes (½ Lesvos and ½ Chios crossbreed) were selected to be of similar body weight (55 kg on average) and similar age (second parturition, 24-month-old on average). These ewes were kept in a feedlot and fed ad libitum a conventional diet (Control diet), based on alfalfa hay, straw, and concentrate feed that contained soybean meal for a period of thirty days (Days 1–30 of the trial). Then, beginning on Day 31 of the trial and for a period of two months (Days 31–90 of the trial), the same ewes were fed a different concentrate feed with 50% less soybean meal that was substituted equally by flaxseed and lupins (Experimental diet). The two diets are presented in Table 1.

#### 2.1.2. Milk Collection

Milk samples from all animals were collected at Days 30 (Group A), 60 (Group B) and 90 of the trial (Group C). The milk of each collection was stored in a small, mobile refrigerated bulk tank (1–4 °C) and transported immediately to a nearby cheese factory (“Tyrokam SA, Cheese Factory”, Amfilochia, Aitoloakarnania, Greece). Three separate Kefalograviera cheesemaking processes were carried using these three milk samples. From each milk sample, 40 L of milk were selected and processed to create 6 wheel-shaped pieces of Kefalograviera (1.1 kg each).

#### 2.1.3. Kefalograviera Cheese Production

The sheep’s milk was initially stored (1–4 °C) in the cheese factory. The next day, the milk was preheated to 35 °C and then passed through a filter before pasteurization. The milk was pasteurized at 69 °C for 10 min and then cooled to 40 °C. After pasteurization, the milk was transferred to the cheesecake to coagulate. Initially, a lactic acid starter culture (a specific blend cultures of *Lactococcus lactis* subsp. lactis, *Lactococcus lactis* subsp. cremoris, Streptococcus thermophilus, Lactobacillus bulgaricus and Lactobacillus rhamnosus) was direct inoculated and subsequently, after 15 min the rennet (a complex set of chymosin and pepsin—95% and 5%, respectively) was added. Once the curd reached the required consistency, it was divided into small 2 cm^3^ cubes and reheated to 48 °C. The curd was then transferred to perforated moulds and pressed into special presses to expel the whey. After the pressure, the cheeses remained in the moulds until the next day, when they were immersed in a tank with a temperature jump of 12–14 °C and a salt content of 18–20% where they were stored for 3 days. The Kefalograviera cheeses were then transported to the maturation room where dry salting was applied for 13 days, at 18 °C and 80% relative humidity. Finally, the cheeses were packaged and placed in a refrigerated chamber with a temperature of 1–4 °C, where they remained for 6 months ripening.

### 2.2. Microbiological Analysis of Kefalograviera Cheese-Cultured Methodology

All cheese samples were cut aseptically and collected from the inside part of the cheese; this being the mass for analysis (cheese mass). Twenty grams of each sample were analysed by cultured microbiological techniques. According to an established protocol, each sample was homogenized with 180 mL of buffer peptone water in a Stomacher bag mixer, at 25 °C, for 1 min, at 24,000 rpm [24]. This was followed by decimal dilutions with buffer peptone water for each sample. After all, 0.1 mL of each dilution was inoculated in nutrient agars. Total Viable Counts (TVC) were enumerated on Plate Count Agar (PCA) after being incubated at 30 °C for 48 h, *Enterococci* and *Streptococci* on Kanamycin Aesculin Azide agar (KAA) at 37 °C for 24 h, *Lactococci* on M17 agar anaerobic at 30 °C and 37 °C for 48 h, *Lactobacilli* anaerobic on MRS agar at 30 °C, 37 °C and 42 °C for 48 h, *Enterobacteriaceae* on Mac Conkey agar at 37 °C for 24 h, and *Staphylococcaceae and Pseudomonadaceae* on Blood Agar at 37 °C for 24 h. All isolates from different culture mediums (M17 agar, MRS agar and KAA agar) were subjected to morphological examination under a microscope, Gram-staining and a catalase test, and were identified as lactic acid bacteria by MALDI-TOF MS technology. The results are expressed as colony-forming units (CFU/g).

#### Identification of Microbiota by MALDI-TOF MS

The identification of microorganisms was performed by using the mass spectrometer MALDI-TOF MS (Bruker Daltonics, GmbH, Bremen, Germany). For the extraction method, according to the manufacturer’s instruction the following steps are essential: using a 1 µL inoculation loop, we transferred the isolated colonies from the nutrient plate into the HPLC-water. Then, 900 µL of pure ethanol were added and mixed. This was followed by centrifuging for two minutes at 13,000–15,000 rpm. Removing the supernatant, we repeated the centrifuging and again the extra ethanol was removed. Then, air-drying the sample for 5 min followed, at 25 °C, adding 25 µL 70% (*v*/*v*) formic acid (for resuspending) and 25 μL pure acetonitrile to the bacterial cell pellet by pipetting. After centrifuging for 2 min, at 13,000–15,000 rpm, 1 µL of the supernatant was placed on the MALDI target plate. Next, air-drying the MALDI target plate at 25 °C followed and 1 μL of BTS QC (Bacterial Test Standard, containing the strain Escherichia coli DH5) was placed on the assigned positions. Finally, the target plate was ready for the mass spectrometer after air-drying from the overlaying of 1 μL HCCA matrix solution onto each sample. Furthermore, after the MALDI-TOF process, all the identification results were obtained per sample and each isolated strain was preserved in vials in a deep freeze. For dendrogram hierarchical clustering, BioTyper software was used [20,25]. Samples’ mass spectra were compared with the BioTyper database reference profiles using the default steps (Bruker Daltonics, GmbH, Bremen, Germany).

The analysis was carried out as per the standard operating procedure for the instrument and built-in software. The raw spectra obtained for the replicates were pre-processed by smoothing and baseline subtraction using Flex Analysis software followed by verification of the quality of each spectrum measurement. The processed selected spectra were then used to create the Main Spectra Projection (MSP) using the automated MSP creation functionality included in MALDI Biotyper 3.0 software. The MSP contains all the information about the mean peak masses, mean peak intensities and mean peak frequencies. These MSPs for each strain were then fed to the functionality of the dendrogram (MALDI Biotyper Compass Explorer), to carry out analysis and produce graphs.

### 2.3. Chemical Analysis

The cheese samples were analysed for fat, moisture, protein and salt using a near-infrared spectrometer FoodScan (FOSS, Electric A/S, Hillerød, Denmark) in transmittance mode. Samples were acclimated at room temperature (20 °C) before analysis. Each cheese sample was measured in triplicate. Cheese acidity was measured by a portable pH meter Sentron 1001 (Sentron Europe, Roden, The Netherlands) [26].

#### 2.3.1. Determination of the Kefalograviera Cheese Fatty Acids Composition

Fatty acid methyl esters (FAMEs) were prepared from the Kefalograviera cheese samples according to the Bligh and Dyer [27] method and the International Organisation for Standardisation ISO [28] as has been reported previously by Papaloukas et al. [29]. Analysis of FAMEs was performed on an Agilent Technologies 6890N gas chromatograph (GC), equipped with a flame ionization detector (FID) and a DB-23 capillary column (60 m × 0.25 mm i.d., 0.25 μm film thickness). Each peak was identified and quantified using a 37 component FAME mix (47885-U Supelco, Sigma-Aldrich, St. Louis, MO, USA) and the PUFA-2, Animal source (47015-U Supelco, Sigma-Aldrich, St. Louis, MO, USA). After analysis, the saturated fatty acids (SFA), monounsaturated fatty acids (MUFA), polyunsaturated fatty acids (PUFA), unsaturated fatty acids (UFA), n-3 PUFA and n-6 PUFA were further grouped together.

The Index of Atherogenicity (AI) and the Index of Thrombogenicity (TI) were determined as suggested by Ulbricht and Southgate [30] and Paszczyk and Łuczyńska [31]:

- AI = (C12:0 + (4 × C14:0) + C16:0)/(Σn-3 PUFA + Σn-6 PUFA + Σ MUFA).

The Index of Thrombogenicity (TI) was calculated as

- TI = (C14:0 + C16:0 + C18:0)/((0.5 × C18:1) + (0.5 × other MUFA) + (0.5 × Σn-6 PUFA) + (3 × Σn-3 PUFA) + Σn-3 PUFA/Σn-6PUFA).

Hypocholesterolaemic fatty acids (DFA), hypercholesterolaemic fatty acids (OFA) and the H/H index (DFA/OFA) were calculated according to Paszczyk and Łuczyńska [31] and Osmari et al. [32]:

- DFA = UFA + C18:0;
- OFA = C12:0 + C14:0 + C16:0;
- H/H index = DFA/OFA.

#### 2.3.2. Determination of Kefalograviera Cheese TBARs

For the TBARs determination, samples of Kefalograviera cheese were processed (one from each group), using the method described by Papastergiadis et al. [33]. The absorbance was measured at 532 nm against a blank sample using a UV-VIS spectrophotometer (UV-1700 PharmaSpec, Shimadzu, Japan). Results were expressed as mg MDA (Malondialdehyde) per kg of Kefalograviera cheese.

### 2.4. Statistical Analysis

The data for the Kefalograviera samples were analysed using the SPSS statistical package (Version 20, IBM SPSS, Armonk, NY, USA). The three groups were used as independent variables. Each “wheel-shaped piece” of Kefalograviera was considered a replication for each group. Microbiology data were log-transformed (log10) prior to analysis. Data homogeneity was tested using Levene’s test. One-way ANOVA and Tukey’s post-hoc tests were used to compare the means of the three groups. A Kruskal–Wallis test was applied for data that were not homogeneous. A Mann–Whitney test was applied for pairwise comparison of data in the microbiology analysis. The significance level was set at 5% (*p* < 0.05) for all tests.

## 3. Results

The characterization of the Kefalograviera cheese microbiota was performed in this study, aiming to determine the potential differences associated with the effects of sheep fed with an omega-3-enriched diet. The results of the microbiological analysis are given in Table 2. No pathogenic bacterial species were recorded in the Kefalograviera cheese in all samples.

**Figure 1:**
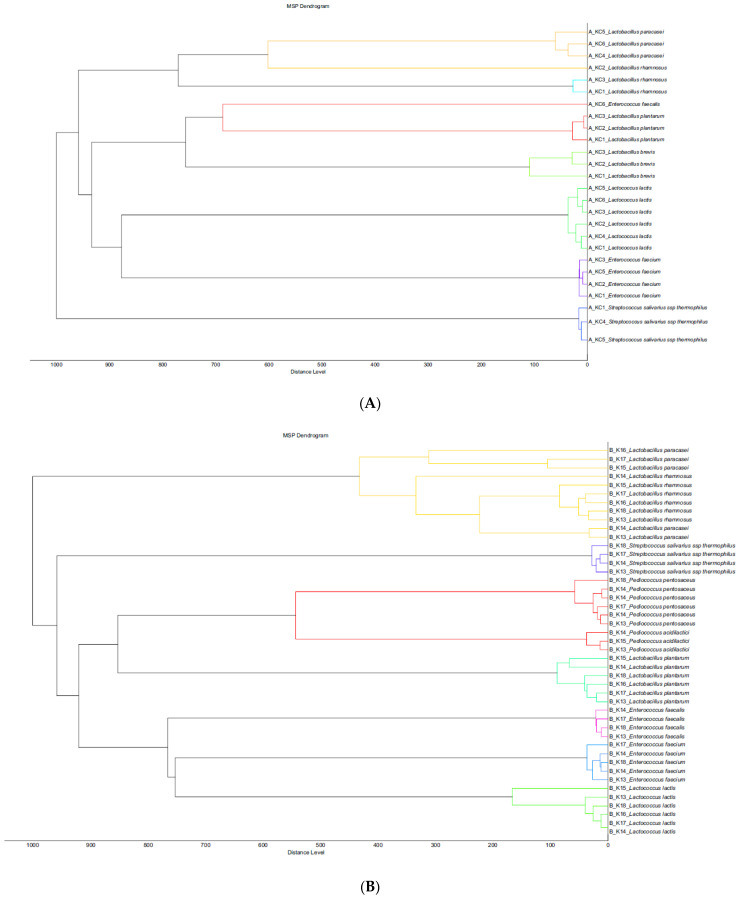

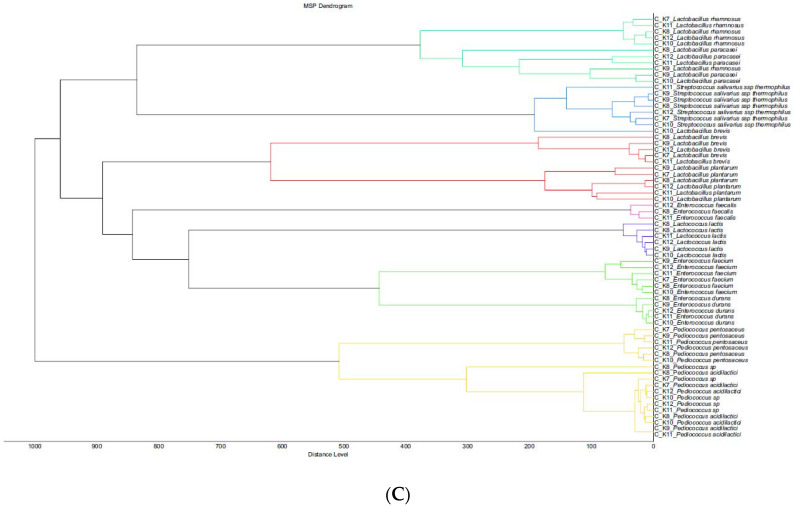
(**A**–**C**) Phylloproteomic trees (dendrograms) of the investigated bacterial species based on the MSP identification via the MALDI Biotyper platform for the three groups A, B and C. The dendrogram’s scale on the bottom represents the relative distance used in the clustering analysis.

**Figure 2:**
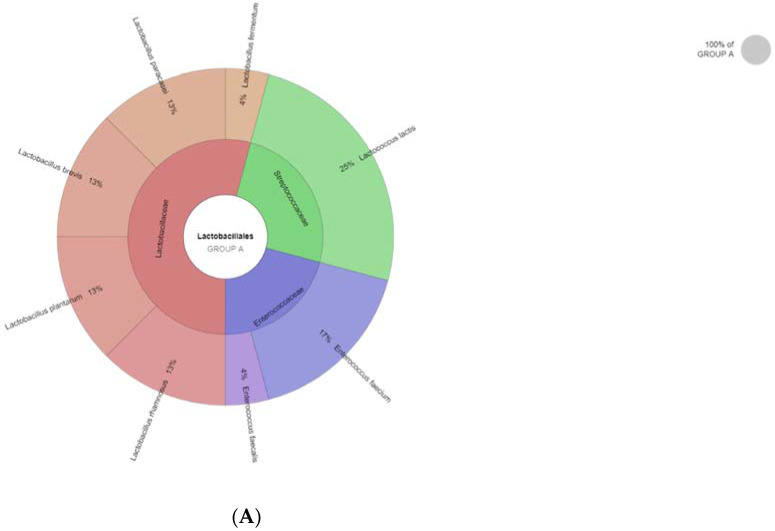

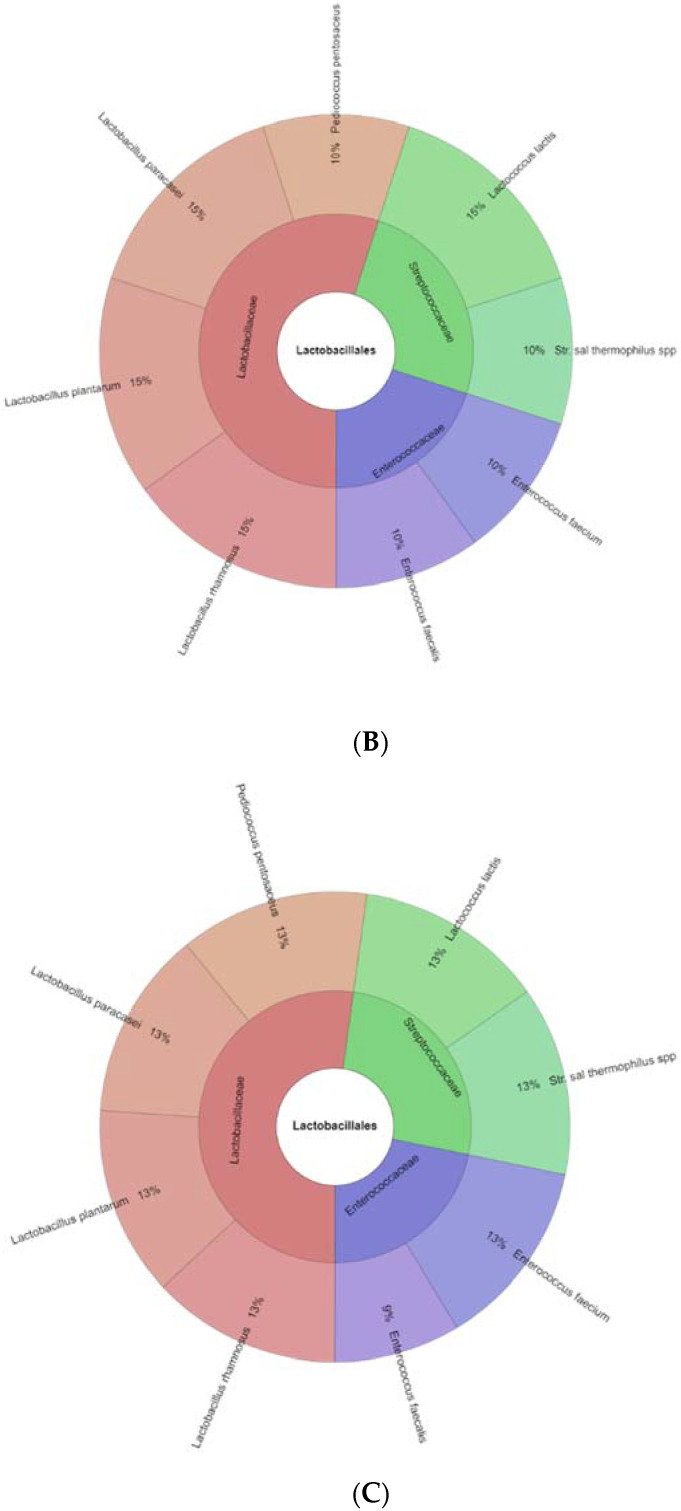
(**A**–**C**) Krona charts for the three groups A, B and C, automatically showing the taxonomic identification and relative abundance of bacterial species (outermost ring: species; middle ring: family; innermost ring: order).

Figure 1A–C represent the phylloproteomic trees (dendrograms) of the investigated bacterial species based on the MSP identification via the MALDI Biotyper platform for the three groups A, B and C. The dendrogram’s scale on the bottom represents the relative distance used in the clustering analysis. Figure 2A–C represent the Krona charts for the three groups A, B and C, automatically showing the taxonomic identification and relative abundance of bacterial species (outermost ring: species; middle ring: family; innermost ring: order).

The phylloproteomic relationship between the isolates presented in the MSP dendrograms revealed the presence of 7 clusters of closely related bacterial species for Groups A, B and C, respectively. These MSP-derived dendrograms illustrate the hierarchical clustering and provide information concerning the relationship status between the isolates at the genus but also species level. Distances between species are calculated as a percentage of spectra similarity. The results of the MALDI-TOF MS identified 140 isolates in total at the species level. According to the krona charts (Figure 2A–C), the microbial diversity in the cheese samples can be estimated, originating from the three groups of the experiment. The LAB species, which were found in both groups of cheeses (Table 2), were isolated in different counts. *Lactococcus* spp., *Lactobacillus* spp., *Enterococcus* spp. and *Pediococcus* spp. were the most abundant taxa identified among all the cheese samples (Figure 1A–C). Microbial composition and diversity differ from cheese group A to cheese groups B and C, while greater homogeneity was apparent in cheeses of Groups B and C. *L. lactis* was present in the highest percentages in most of the cheese samples, ranging from 21% to 14% to 10% of the relative abundance in Groups A, B and C, respectively. *Streptococcus sal* sp. *thermophilus* was present in all samples as well, with values ranging from 10% (Group B, C) to 14% (Group A). An increase in the abundance percentage of specific bacterial species, such as *L. paracasei*, *Lactobacillus plantarum* and *L. rhamnosus*, was observed at the cheese samples of Group B, in contrast with the cheese samples of Groups A and C. Kefalograviera cheese of Group B had a lower (*p* < 0.001) *L. rhamnosus* count, compared to Groups A and C. Moreover, Kefalograviera cheese of Group B had a lower (*p* = 0.009) *L. paracasei* count compared to Group C. Species such as *Pediococcus acidilactici* and *Pediococcus pentosaceus* were identified only in experimental cheese samples (Group B and C), with the second forming an increase in its percentage to 10% in Group C.

Table 3 presents the fatty acid profile of the Kefalograviera cheeses. Oleic acid (C18:1) was the most abundant fatty acid, followed by palmitic acid (C16:0), stearic acid (C18:0) and myristic acid (C14:0). Capric acid was decreased (*p* = 0.015) in Groups B and C, compared to Group A. Undecanoic acid was increased (*p* = 0.031) in Group C compared to Group A. Alpha-linolenic acid was highest (*p* = 0.001) in Group C, followed by Group B and then Group A. Total polyunsaturated omega-3 fatty acids were highest (*p* = 0.001) in Group C, followed by Group B and then Group A. The ratio of omega-6 to omega-3 fatty acids was higher (*p* = 0.003) in Groups B and C, compared to the control Group A. The calculated health indexes AI, TI, DFA, OFA and H/H did not differ (*p* > 0.05) between the groups and were within the expected limits.

Twenty-three different fatty acids were detected in the Kefalograviera cheese samples, common in all three groups (Table 3). Their carbon skeleton ranged from six (6) molecules to twenty (20) molecules. From these fatty acids eleven (11) were saturated (SFA) and twelve (12) were unsaturated (UFA). From the UFA, seven (7) were monounsaturated (MUFA) and five (5) were polyunsaturated (PUFA). The total content of SFA was greater than UFA, while the MUFA content was greater than PUFA, a typical order reported by most researchers [34]. Significant differences in the fatty acid content of the cheeses were noted for some saturated fatty acids (Capric acid and Undecanoic) and the polyunsaturated alpha-linolenic acid in the omega-3-supplemented diet. A significant increase in total omega-3 fatty acids and decreased ratio of omega-6 to omega-3 fatty acids was also observed for the omega-3-supplemented diets, especially at the cheese from the last sampling of the trial. It should be noted that the ratio of omega-6 to omega-3 for Group C was above the health limit, which is reported to be 4:1 [35].

Results of the chemical composition of the cheese samples are shown in Table 4. The total solids of Group B were found to be lower (*p* = 0.041) compared to the control Group C. Salt was lower (*p* = 0.016) in Groups B and C, compared to the control. The other examined parameters did not differ. Moreover, the lipid oxidative markers (TBARs) were similar and within acceptable ranges.

## 4. Discussion

Animal dietary protocol is the factor of paramount importance that affects the flavour and the other organoleptic characteristics of cheese. Undoubtedly, the authenticity and identity of a product such as cheese depends on the microbiota, which ferments the substrates that provides nutrition. Fatty acids play a critical role in the physical and chemical status of a cheese, yet it is not clear how they affect the microbiota or how they are affected by the bacterial populations [36,37].

### 4.1. Microbial Dynamics in Kefalograviera Samples

Microbiota is a key factor responsible for all sensory characteristics and product quality and may be beneficial in the case of probiotic microorganisms or harmful in the case of spoilage and pathogenic microorganisms [20,21,38]. Although we searched thoroughly for research papers on the effect of increased dietary omega-3 fatty acids on the microbiota of Kefalograviera cheese, we could not locate any. Hence, this paper is, if not the first, among the first negotiating this issue, and has a strong element of novelty.

In our study, the predominant microbial populations in all three groups of cheeses were the lactic acid bacteria (LAB) ones. This was an expected finding since these microorganisms contribute to the development of the taste and texture of cheese, while some species produce bacteriocins that contribute to their conservation by inhibiting the growth of antagonistic pathogenic and spoilage microorganisms [39]. The most important role of the *L. lactis* strains in cheese production is their ability to rapidly produce lactic acid, which promotes the coagulation and formation of curd. Another essential characteristic is the possession of a proteolytic enzyme system enhancing the development of cheese flavour [40]. Next to these technical aspects, a number of metabolites contribute to the safety of cheese or suppress spoilage [40]. *Lactobacillus* spp. grows at a very low rate during the initial weeks of cheese ripening but may dominate the cheese microbiota during ripening. These bacteria are important both in the processing of fresh and ripened cheeses during the ripening process, as they release bioactive peptides, vitamins, and oligosaccharides [41].

*L. paracasei* can also be used as starter bacteria, isolated in both starter cultures and unripened cheeses. It is similar to other strains of *Lactobacillus* bacteria that appeared in our cheese samples, such as *L. rhamnosus*, whose presence can be in both the starter and non-starter cultures added for Kefalograviera production [42]. *L. paracasei* exerts probiotic action by enhancing the immune system and improves gut microbiota by reducing harmful bacteria and boosting the numbers of beneficial bacteria, such as Bifidobacteria or *Lactobacilli*. It was isolated from both experimental groups as well as from the control group, although in the latter group it was present in half of the samples. Its counts differed significantly (H = 10.881, *p* = 0.004) among the existing groups but since the Group B counts were lower than the ones of the control, these differences cannot be attributed to the omega-3 dietary content. Interestingly, the same conclusion is also valid for *L. rhamnosus* because it shows the same distribution as *L. paracasei.* Its counts were significantly different among the groups (H = 11.2301, *p* = 0.004) but the Group B counts were lower than the ones of the control group.

*L. brevis* may represent the most common adventitious non-starting LAB found in cheeses made from pasteurized milk [43]. The bacterium was also present in Kefalograviera produced with or without starter culture and originated from milk of animals fed with flaxseed or lupins but not from milk of sheep fed with soybean, with the counts not being statistically significant (U = 6, *p* = 0.066). *L. brevis* may produce CO_2_, causing gas openings in packaged cheeses [44]. *L. plantarum* has been isolated from many Greek cheeses representing non-starter LAB bacteria, and some of its strains can be used as probiotics [45,46,47]. In the present study, this species was isolated from all groups but in statistically significant different counts (H = 8.5526, *p* = 0.014), escalating with respect to the dietary content of omega-3 fatty acids. As already mentioned, *Lc. lactis* is vital for cheese manufacturing and can be used quite often as a single culture or mix starter culture because during ripening lipolytic and proteolytic enzymes released by the bacterium enhance the taste and the aroma mainly in hard cheeses. In our cheeses, *Lc. lactis* appeared in all samples of cheeses of all groups regardless of its use or not as part of the starter culture [48], but its counts showed no significant difference among the groups (H = 3.0906, *p* = 0.213). The non-starter lactic acid bacteria constitute a crucial part of the hard type cheeses’ microbial population. In hard and semi-hard types of cheeses, such as Kefalotyri, Kefalograviera, Kashkavali and Parmesan, bacterial species such as *Pediococcus* spp., *L. casei, L. paracasei, L. brevis, L. plantarum, L. rhamnosus* and *Lc. lactis* have been used in starter mixtures to develop the desired amount of lactic acid, to affect flavour and to accelerate ripening [46,49,50].

*Pediococcus* spp. belongs to the Lactobacillaceae family and is associated with cheese fermentation, either as indigenous microbiota or as starter culture [47,51]. However, *Pediococci* are non-starter LAB bacteria in cheeses such as white-brine and hard yellow cheeses, having the ability to produce bacteriocins, which are termed as antilisterial [52]. In our study, *P. pentosaceus* and *P. acidilactici* strains were isolated from the cheeses of both experimental groups. Both species were present in all cheeses from Group C. In cheeses from Group B, however, *P. pentosaceus* was present in 4 out of 6 samples, and whole *P. acidilactici* was present in 2 out of 6 samples. This finding reveals an escalation of the prevalence of both species with respect to the omega-3 content of the diet and suggests that it can be considered as reasonably attributable to the increased content of the dietary omega-3 fatty acids. This escalation affected only the prevalence of the bacterial species involved and not their counts, which were not significantly different (*p* = 0.06576 for *P. pentosaceus* and *p* = 0.37886 for *P. acidilactici*). Finally, another group of isolates of *Pediococci* (*Pediococcus* spp.) were isolated only from cheeses made of milk from Group C, indicating that the enriched omega-3 dietary content favoured the growth of this species.

The genus *Enterococcus*, such as other LAB, has also been featured in dairy industry for decades due to its specific biochemical traits such as lipolysis, proteolysis and citrate breakdown, hence contributing the typical tastes and flavours to dairy foods. Furthermore, the production of bacteriocins by enterococci (enterocins) is well documented. These technological applications have led to propose enterococci as adjunct starters or protective cultures in fermented foods. Moreover, enterococci are nowadays promoted as probiotics, which are claimed for the maintenance of normal intestinal microflora, stimulation of the immune system and improvement of nutritional value of foods. At the same time, enterococci present an emerging pool of opportunistic pathogens for humans as they cause disease, possess agents for antibiotic resistance and are frequently armed with potential virulence factors [53]. Because of this “dualistic” nature, the use of enterococci remains a debatable issue. However, based on a long history of safe association of particular enterococci with some traditional food fermentations, the use of such strains appears to bear no particular risk for human health [54].

*E. faecalis, E. faecium* and *E. durans* were also present in the produced cheese Kefalograviera samples. *E. durans* was isolated only from samples of Group C, while the other two *Enterococci* were isolated from all groups but in different prevalence rates. *E. faecium* was isolated from 4 out of 6 samples of Group A, from 4 out of 6 samples of Group B and from all samples of Group C. In turn, *E. faecalis* was isolated from 1 sample out of 6 of Group A, from 4 samples out of 6 of Group B and from 4 samples out of 6 of Group C. For every species the differences in their counts among the groups were not significant (*p* = 0.472 *E. faecium* and *p* = 0.129 for *E. faecalis*). Such findings suggest a moderate impact of the dietary content of the omega-3 fatty acids on the *Enterococci*. The *Enterococcus* species are common commensal bacteria inhabiting the gastrointestinal tract of the animals, frequently isolated in dairy or other food products. Since they have a potential for thermophilic fermentations they can be used as cheese starter cultures or probiotics. *Enterococci* are widely distributed in raw and pasteurized milk cheeses having a significant role in flavour development but also as a potential pathogen as the natural habitat of enterococci is the mammalian intestine. *E. faecalis* is dominant in human and animal faeces but *E. faecium* has also been found [55]. It is possible that *Enterococci* as members of the intestinal microflora of the sheep and other animals living in the farm, contaminated the milk and the produced cheeses. Many researchers have reported the isolation of *Enterococci* strains from traditional yogurts and other dairy products [47,56,57]. *Enterobacteriaceae* family bacteria were recorded in the cheese from sheep which consumed flaxseed and lupines. It is an interesting finding, considering that these cheeses contained increased the levels of PUFAs and particularly omega-3 fatty acids, which have a well-known antibacterial action [58,59]. It is possible that the isolated strains were resistant to the n:3 PUFAs. *S. sal.* sp. *thermophilus* was present in all groups but its presence in all the samples of Group C marked its higher prevalence rate while its counts among the three groups were not significantly different (*p* = 0.213).

In our study, no statistically significant differences in total viable bacteria count and in Coagulase Negative Staphylococci (CNS) counts were recorded in the milk produced by sheep fed either soybean or flaxseed and lupines (*p* = 0.31784). All CNS species were isolated from the control group, suggesting perhaps a protective role of omega-3 fatty acids to the udder health, given the fact that these bacteria are the major cause of subclinical mastitis in small ruminants [60,61,62].

By comparing the isolated species and their counts in the three different groups, it is rather obvious that the core microbiota of the cheese was overall affected by the dietary enrichment in omega-3 fatty acids. The exact biological mechanism by which this is achieved is not yet clear and further research is needed although we suspect that it must be somehow connected to the metabolic pathways of these acids in the matrix of fermented milk. This impact can affect directly the bacterial cells and the dynamic balance of the bacterial populations. The latter is supported by our findings (Table 2), which show, e.g., an increase in the case of *Lactococcus lactis* counts in Group B with respect to the other two groups, while a decrease in the counts of *Lactobacillus rhamnosus* in Group B with respect to the other two groups is observed. These findings show a nonlinear relationship, while in the case of other species, such as *Lactobacillus plantarum*, the relationship between the omega-3 dietary content and bacterial counts is linear (they increase significantly from Group A to Group C).

### 4.2. Chemical Composition of the Cheeses—TBARs Values

The crude chemical composition of the cheeses among the three groups was not affected significantly by the different feeding treatments. The protein and fat content as well as the pH remained unaltered. The salt content and the total solids showed a statistically significant difference (*p* = 0.016 for the salt and *p* = 0.041 for the total solids), these differences being 2.52% for total solids and 0.08% for salt. It could be hypothesized that these differences can be attributed to the process of syneresis where different amounts of water in the curd can be lost along with salt and minerals.

TBARS are by-products of lipid oxidation. Malondialdehyde (MDA) is such a by-product, which reacts with thiobarbituric acid. The complex can be photometrically measured and hence the lipid oxidation can be assessed. This assessment must be cautious though because MDA is not the only product, and neither is exclusively produced through lipid oxidation. TBARS in cheese have been associated with inferior quality and organoleptic problems [63,64]. Zamoga and Hidalgo (2005) claim that increased TBARS are responsible for the colour alteration (browning) of the cheese due to reaction with amino acids and proteins [65]. In the present study, the TBARS were generally low and within acceptable ranges and not significantly different among groups. This is in accordance with the results of Fusaro et al. (2020) who also found no differences in TBARS concentrations in cheeses in a very similar experiment [66]. This finding is an expected one, since the overall fat content was not significantly different among groups, implying that similar levels of fat oxidations occurred in the curds.

### 4.3. Fatty Acid Content of the Cheeses

Twenty-three different fatty acids were detected in the Kefalograviera cheese samples, common in all three groups (Table 3). Their carbon skeleton ranged from six (6) molecules to twenty (20) molecules. From these fatty acids eleven (11) were saturated (SFA) and twelve (12) were unsaturated (UFA). From the UFA, seven (7) were monounsaturated (MUFA) and five (5) were polyunsaturated (PUFA). The total content of SFA was greater than UFA, while the MUFA content was greater than PUFA—a typical order reported by most researchers [34]. Significant differences in the fatty acid content of the cheeses were noted for some saturated fatty acids (Capric acid and Undecanoic) and the polyunsaturated alpha-linolenic acid in the omega-3-supplemented diet. A significant increase in the total omega-3 fatty acids and decreased ratio of omega-6 to omega-3 fatty acids was also observed for the omega-3-supplemented diets, especially at the cheese from the last sampling of the trial. It should be noted that the ratio of omega-6 to omega-3 for group C was above the health limit, which is reported to be 4:1 [35].

The fatty acid content of milk depends on factors such as the diet, the breed of the animals, their age, their lactation stage and their body condition and various environmental sources of stress, such as extreme temperatures and grazing vegetation [11,67]. The epithelial cells of the mammary gland synthetize and secrete fatty acids. The short-chain fatty acids (C4:0–C14:0) and a portion of C16:0 is synthetized de novo in the secretory cells from acetate and betahydroxycarbonate, which are products of the rumen fermentations. The long-chain fatty acids (C18:0–C22:0) are synthetized by lipids that are transported to the mammary gland via blood circulation and have been absorbed in the small intestine or have been mobilized from the adipose tissue [68]. Nutrition plays a crucial role since it can significantly affect the content of omega-3 fatty acids and pastures are an excellent source of these substances as well as various oils of plant or marine origin. Rumen fermentations represent a serious hazard due to increased biohydrogenation that render the unsaturated fatty acids to saturate. It seems that in goats and sheep, the rate of rumen evacuation is faster than in cows and therefore a larger portion of dietary unsaturated fats escapes biohydrogenation in small ruminants. Hence, cheeses originating from small ruminants’ milk are more beneficial to the consumer. In a previous study conducted on the same area, the major fatty acids (FAs) in ovine milk were found to be palmitic and oleic acids [69]. The importance of lipids in sheep milk lies not only in their high nutritional value but also to their effect in the taste and flavour as well as in the manufacturing properties of the dairy products [70,71]. In comparison to the other ruminants’ milk, sheep milk has higher total fat and conjugated linoleic acid (CLA) content [72,73]. Factors affecting quantitatively and qualitatively the fatty acid content of milk can be classified as genetic (breed, genotype), physiological (age, stage of lactation, season) and environmental (feeding, grazing) factors and, of course, their interactions [34,67]. Milk fat is characterized by complexity since it contains more than 400 fatty acids [15]. These fatty acids have different numbers of carbon atoms in their molecules (in odd and even numbers), forming straight or branched chains with *cis* and *trans* configurations. Their degree of saturation also varies from saturated (SFA) and monounsaturated (MUFA) to polyunsaturated (PUFA) [15].

In our study, the total PUFAs increased significantly from Group A to Group B by a factor of 4.5, and from Group A to Group C by a factor of 7.7 (Table 3). These findings suggest that the dietary increase in omega-3 fatty acids resulted in an increase in omega-3 in the final product and answer in a positive way one of the aims of this study.

### 4.4. Health Indexes

Most consumers want their diet to be as healthy as possible. Among other determinants of the term “healthy diet” factors such as the content of the diet in saturated and unsaturated fatty acids are critically important due to the fact that many serious and detrimental ailments, such as cardiovascular diseases and certain types of cancer, are associated with the quality of the daily fatty acid intake. To evaluate the health status of a food from its lipid content, certain indexes are calculated. In the present study, the AI index was found to be 1.66 for the control Group A, 1.75 for Group B and 2.24 for Group C. Moreover, the TI index was found to be 2.53 for the control Group A, 2.21 for Group B and 2.38 for Group C. The AI and TI reflect the potential adverse effects of single fatty acids on consumers’ health by increasing the risk of atheroma and thrombus formation. AI describes the ratio between the main categories of saturated fatty acids and the unsaturated fatty acids. TI values represent the analogy of prothrombogenic and antithrombogenic fatty acids, which is the ratio between SFA and MUFA n-3 and PUFA n-6. Numerically speaking, the higher these indexes are the more atherogenic and thrombogenic the cheese is, while when these index values are low, the cheese may exert a beneficial effect to human health [37]. On the other hand, neither human diet is based exclusively on cheese, nor do they exist in artisan production “low fat” dairy products (in the commercial sense of the term). Cheeses with increased atherogenic and thrombogenic indexes do not necessarily cause heart disease or any other ailment, provided of course that the consumers follow a balanced diet and exercise regularly [30]. Indexes relevant to cholesterolaemic fatty acids were also determined in the present trial. DFA was 51.46 for the control Group A, 50.50 for Group B and 44.44 for Group C; OFA was 38.91 for the control Group A, 39.29 for Group B and 43.92 for Group C; the H/H index was 1.323 for the control Group A, 1.285 for Group B and 1.012 for Group C. Increased daily intake of saturated fatty acids leads to an increase in blood cholesterol values, which, in turn, increase the risk for cardiovascular diseases [39]. The length of the carbon chain of saturated fatty acids is the critical factor determining the effect of SFA on serum cholesterol. The different fatty acids affect in a different way the metabolism of lipoproteins. Lauric acid (C12:0), myristic acid (C14:0) and palmitic acid (C16:0) increase the LDL and HDL cholesterol while stearic acid has a neutral effect [46,74]. There is a consensus in the scientific literature that the ruminant diet could affect positively the content of the cheese in beneficiary fatty acids with respect to the consumer’s health [46,47,75,76]. However, the extensive biohydrogenation that takes place in the rumen potentially destroys large amounts of the unsaturated fatty acids of the diet. It is also possible that the bioavailability of these UFAs in the intestine decreases through interaction with other substances such as dietary fibre.

## 5. Conclusions

The bacterial microbiota of the Kefalograviera cheese was overall affected by the dietary treatments enriched in omega-3 fatty acids. This effect did not include all the involved bacterial species and concerned either differences in the prevalence of species (e.g., Pediococci and Enterococci) in the different groups or differences in the populations’ size (the counts; e.g., *L. paracasei* and *L. rhamnosus*). This shift in bacterial populations could lead to the development of strains with increased probiotic or technological potential, thus increasing the health benefit of the consumers. Sheep fed with diets containing a higher content in omega-3 fatty acids produced cheeses with a higher content of omega-3 PUFAs. Dietary linseed and lupins improved the content of PUFAs and decreased the ratio of omega-6/omega-3 (from 18.6/1 to 4.1/1) in Kefalograviera cheese. Cheeses from Group C (sheep fed an omega-3-rich diet for a longer period of time) had a higher content in omega-3 fatty acids than the ones from Group B, which, in turn, had a higher content of omega-3 fatty acids with respect to the controls. Although the examined health indexes (AI, TI, DFA, OFA H/H) of the cheeses did not differ between the experimental groups and the controls, and the chemical composition of the cheeses remained unaltered among the groups in its basic ingredients (proteins and fats), the increase in PUFAs is a promising finding, as Kefalograviera cheese by this manner can promote human health.

## Figures and Tables

**Table 1:** Daily ingredient allowance and chemical composition of the diets offered to dairy ewes during the trial.

Ingredients (on Fresh Weight Basis)	Control Diet (g/Day/Ewe)	Experimental Diet (g/Day/Ewe)
Lucerne hay	1.200	1.200
Barley straw	300	300
Corn grain	540	590
Barley grain	330	150
Wheat bran	180	110
Sunflower seed meal (36% crude protein)	30	180
Soyabean meal (47% crude protein)	300	150
Cotton seed	30	80
Flaxseed	-	75
Lupin seed	-	75
Molasses	20	20
Premix ^1^ with vitamins and inorganic minerals	60	60
Total dry matter intake/day	2630	2640
**Chemical Analysis (%)**		
Dry matter	87.4	87.5
Crude protein (N × 6.25)	16.1	16.1
Ether extract	2.82	4.01
Neutral detergent fibre	33.8	35.1
Acid detergent fibre	19.5	19.9
Acid detergent lignin	4.51	4.98
Ash	6.55	6.52
Starch	18	16.1
**Calculated analysis**		
Calcium	11.5	11.6
Phosphorus (total)	3.82	3.98
PDI g/kg DM	89.4	87.5
PDIA g/kg DM	44.3	42.7
UF_L_	0.71	0.71

^1^ Vitamin and mineral mix contained per kg DM of concentrate: 8000 IU of vitamin A; 90 mg of vitamin E; 3000 IU of vitamin D_3_; 1.5 mg/kg biotin; 6 mg/kg niacin; 45 mg/kg choline; 0.2 mg Co; 3 mg I; 100 mg/kg Fe; 50 mg Mn; 0.45 mg Se; 150 mg Zn; 6 g of NaCl; 4 g of sulphur; 10 g of magnesium oxide; 15 g of monocalcium phosphate and 21 g of limestone. PDI = protein digestible in the small intestine; PDIA = protein digestible in the small intestine supplied by rumen–undegraded dietary protein; UF_L_ = forage unit for lactation. The chemical composition of the ingredients was based on the Premier Nutrition database [23].

**Table 2:** Distribution of the identified microbial species (log cfu/mL) showing the number of Kefalograviera cheese samples in which each microbial species was identified (6 in total for each group) and the mean counts (±standard deviation).

Isolated Bacteria	Group A Day 30		Group B Day 60		Group C Day 90		
	Control Diet		Experimental Diet (1st Month)		Experimental Diet (2nd Month)		*p*
Total viable counts	6	7.440 ± 0.594	6	7.181 ± 0.620	6	7.767 ± 1.355	0.560
*Lactococcus lactis*	6	6.999 ± 0.569	6	7.522 ± 0.315	6	6.977 ± 0.999	0.327
*Lactobacillus rhamnosus*	3	7.352 ± 0.453 ^b^	6	6.145 ± 0.224 ^a^	6	7.488 ± 0.274 ^b^	<0.001
*Lactobacillus plantarum*	3	6.310 ± 0.084	6	6.805 ± 1.537	6	7.421 ± 0.352	0.310
*Lactobacillus brevis*	3	6.060 ± 0.283	0	-	6	7.036 ± 0.869	
*Lactobacillus fermentum*	1	3.699 ± 0.000	0	-	0	-	
*Lactobacillus paracasei*	3	7.201 ± 1.154 ^ab^	6	6.320 ± 1.104 ^a^	6	8.415 ± 0.662 ^b^	0.009
*Enterococcus durans*	0	-	0	-	6	5.351 ± 0.189	
*Enterococcus faecium*	4	6.128 ± 1.309	4	6.033 ± 0.229	6	5.665 ± 1.254	0.780
*Enterococcus faecalis*	1	6.301 ± 0.000	4	6.070 ± 0.267	4	5.382 ± 0.846	
*Staphylococcus caprae*	1	4.477 ± 0.000	0	-	0	-	
*Staphylococcus haemolyticus*	1	4.699 ± 0.000	0	-	0	-	
*Staphylococcus haulius*	1	3.477 ± 0.000	0	-	0	-	
*Streptococcus sal.* sp. *thermophilus*	4	7.979 ± 0.783	4	8.089 ± 0.182	6	8.431 ± 0.350	0.333
*Pediococcus* spp.	0	-	0	-	6	5.003 ± 0.446	
*Pediococcus pentosaceus*	0	-	4	6.064 ± 0.136	6	6.685 ± 0.777	
*Pediococcus acidilactici*	0	-	2	4.278 ± 0.032	6	4.422 ± 0.481	

^a,b^ Values in the same row without superscripts in common differ significantly (*p* ≤ 0.05).

**Table 3:** Fatty acid profile of the Kefalograviera cheese samples (% of total fatty acids ± standard deviation).

Fatty Acids	Group A Day 30	Group B Day 60	Group C Day 90	
	Control Diet	Experimental Diet (1st Month)	Experimental Diet (2nd Month)	*p*
C6:0	0.71 ± 0.028	1.11 ± 0.325	1.32 ± 0.113	0.115
C8:0	0.62 ± 0.106	1.18 ± 0.856	1.75 ± 0.092	0.226
C10:0	6.61 ± 0.035 ^b^	5.68 ± 0.240 ^a^	5.83 ± 0.071 ^a^	0.015
C11:0	0.11 ± 0.014 ^a^	0.16 ± 0.028 ^ab^	0.32 ± 0.064 ^b^	0.031
C12:0	2.13 ± 0.028	3.4 ± 1.782	4.89 ± 0.304	0.164
C13:0	0.25 ± 0.057	0.25 ± 0.064	0.45 ± 0.049	0.066
C14:0	8.41 ± 0.021	9.93 ± 2.249	11.62 ± 0.078	0.188
C14:1n5	0.87 ± 0.021	0.6 ± 0.382	0.34 ± 0.028	0.198
C15:0	0.91 ± 0.007	0.93 ± 0.071	1.08 ± 0.049	0.067
C15:1n5	0.34 ± 0.014	0.33 ± 0.035	0.25 ± 0.035	0.093
C16:0	28.37 ± 0.573	25.96 ± 1.605	27.41 ± 0.325	0.197
C16:1n7	2.24 ± 0.127	1.58 ± 0.898	0.93 ± 0.014	0.184
C16:1n9	0.43 ± 0.049	0.39 ± 0.071	0.38 ± 0.028	0.698
C17:1n7	0.46 ± 0.035	0.34 ± 0.177	0.27 ± 0.007	0.297
C18:0	12.72 ± 0.071	11.06 ± 2.404	9.24 ± 0.177	0.185
C18:1n9c	30.49 ± 0.601	29.72 ± 5.982	24.26 ± 1.011	0.299
C18:1n7c	0.28 ± 0.028	0.72 ± 0.636	1.19 ± 0.177	0.205
C18:2n6t	0.48 ± 0.001	0.82 ± 0.389	1.02 ± 0.017	0.196
C18:2n6c	2.25 ± 0.007	3.08 ± 1.117	3.88 ± 0.233	0.189
C18:3n6	0.49 ± 0.064	0.93 ± 0.714	1.26 ± 0.17	0.325
C18:3n3	0.16 ± 0.014 ^a^	0.72 ± 0.064 ^b^	1.23 ± 0.071 ^c^	0.001
C20:0	0.26 ± 0.035	0.31 ± 0.085	0.26 ± 0.028	0.653
C20:4n6	0.25 ± 0.014	0.21 ± 0.035	0.19 ± 0.001	0.152
Totals	99.84 ± 0.099	99.41 ± 0.113	99.37 ± 0.057	
Saturated fatty acids—SFA	61.10 ± 0.537	59.98 ± 4.773	64.18 ± 0.941	0.418
Unsaturated fatty acids—UFA	38.74 ± 0.636	39.44 ± 4.660	35.20 ± 0.997	0.383
Mono unsaturated fatty acids—MUFA	35.11 ± 0.707	33.68 ± 6.908	27.61 ± 1.117	0.290
Poly unsaturated fatty acids—PUFA	3.63 ± 0.071	5.76 ± 2.249	7.59 ± 0.120	0.121
PUFA n3 (omega-3)	0.16 ± 0.014 ^a^	0.72 ± 0.064 ^b^	1.23 ± 0.071 ^c^	0.001
PUFA n6 (omega-6)	2.99 ± 0.021	4.11 ± 1.471	5.10 ± 0.219	0.191
Ratio n6/n3 (omega-6/omega-3)	18.66 ± 1.789 ^b^	5.75 ± 1.556 ^a^	4.14 ± 0.064 ^a^	0.003
Index of atherogenicity (AI): (C12:0 + 4 × C14:0 + C16:0)/UFA	1.66 ± 0.035	1.75 ± 0.524	2.24 ± 0.085	0.278
Index of Thrombogenicity (TI): (C14:0 + C16:0 + C18:0)/(0.5 × MUFA + 0.5 × PUFAn6 + 3 × PUFAn3 + n3/n6)	2.53 ± 0.085	2.21 ± 0.339	2.38 ± 0.042	0.442
Hypocholesterolaemic fatty acids (DFA) (UFA + C18:0)	51.46 ± 0.566	50.50 ± 7.064	44.44 ± 1.174	0.323
Hypercholesterolaemic fatty acids (OFA) (C12:0 + C14:0 + C16:0)	38.91 ± 0.523	39.30 ± 5.636	43.93 ± 0.707	0.365
H/H index (Hypocholesterolaemic/Hypocholesterolaemic fatty acids)	1.323 ± 0.033	1.285 ± 0.368	1.012 ± 0.042	0.383

^a,b,c^ Values in the same row without superscripts in common differ significantly (*p* ≤ 0.05).

**Table 4:** Chemical composition and lipid oxidative markers (TBARs) of the Kefalograviera cheese samples (means ± standard deviation).

	Group A Day 30	Group B Day 60	Group C Day 90	
	Control Diet	Experimental Diet (1st Month)	Experimental Diet (2nd Month)	*p*
Moisture (%)	39.17 ± 1.80	40.95 ± 1.21	39.70 ± 1.58	0.157
Total solids (%)	60.85 ± 1.81 ^b^	58.33 ± 1.46 ^a^	60.30 ± 1.58 ^ab^	0.041
Fat (%)	28.03 ± 3.41	26.23 ± 1.24	26.87 ± 1.38	0.431
Protein (%)	27.88 ± 2.71	27.17 ± 1.11	28.82 ± 1.06	0.309
Salt (%)	2.16 ± 0.06 ^b^	2.08 ± 0.02 ^a^	2.08 ± 0.02 ^a^	0.016
pH	4.84 ± 0.09	4.86 ± 0.07	4.79 ± 0.16	0.757
TBARS (mg/kg)	4.2	3.4	3.6	-

^a,b^ Values in the same row without superscripts in common differ significantly (*p* ≤ 0.05).

## Data Availability

The data are contained within the article.
